# 2152: Adipose tissue measurements of computed tomography scan studies as a possible predictor of cancer recurrence after radical prostatectomy

**DOI:** 10.1017/cts.2017.248

**Published:** 2018-05-10

**Authors:** Ana I. Ortiz, Juan C. Jorge, Lourdes Guerrios

**Affiliations:** University of Puerto Rico-Medical Sciences Campus, San Juan, Puerto Rico

## Abstract

OBJECTIVES/SPECIFIC AIMS: The goal of this pilot study is to provide a reliable anatomical algorithm for the measurement of adipose tissue within the pelvic cavity as a predictor of prostate cancer aggressiveness and recurrence after radical prostatectomy. METHODS/STUDY POPULATION: We will conduct a retrospective analysis of men treated with radical prostatectomy between 2012 and 2016 at the VA Caribbean Health Care System. Clinical variables, pathology reports, and computed tomography will be reviewed. Pelvic and periprostatic fat (PF) will be measured to determine association between PF and cancer aggressiveness and recurrence. RESULTS/ANTICIPATED RESULTS: We expect a positive association between PF and cancer aggressiveness and recurrence among patients who underwent radical prostatectomy. DISCUSSION/SIGNIFICANCE OF IMPACT: Measurement of subcutaneous and PF within the pelvic cavity can provide a reliable anatomical measure which can be used as a proxy measure to identify those with higher risk of recurrence and develop better prevention and treatment strategies, especially in Hispanic men.

